# A Few Words to Quackery

**Published:** 1831-01-01

**Authors:** 


### A FEW WORDS TO QUACKERY. By DEMOCRITUS, jun.

THE above is the title of a brochure recently published, and said to be from the pen of an eminent literary physician. We cannot believe it; for a more inane and tiresome, yet conceited, performance, we have rarely perused. Think of a physician at this time of day, with all the follies of St. John Long and his disciples before him, sitting down in the British Museum, and giving us a dull abstract of various pamphlets, published one hundred years ago, respecting the pretended lithontriptic powers of egg-shells! These excerpta, together with a long list (such as appeared at the Old Bailey in favour of Long) of those who believed themselves cured by Mrs. Stephens' nostrum, fill three-fourths of this 12-penny pamphlet. That any man not in Bedlam, but actually in the British Museum, should possess a brain so totally deprived of all claim to sanity, as to put forth such an Essay at this period, would be incredible, were not the fact before our eyes. The author says, in conclusion—"I had intended to write only of St. John Long, but have found myself *insensibly* drawn into giving a history of Mrs. Stephens." For *insensibly* he might have said *senselessly*. We believe that neither LONG nor STEPHENS is worth the trouble of a penny pamphlet; and, therefore, if physicians have nothing else to do than to ransack the library of the British Museum for materials for a brochure, they might employ their literary leisure to much greater advantage. The title is the only cunning part of the performance, being grafted on the excitement of the moment. The following extract is the best specimen we could find in the pamphlet.

"You probably know some nicish girl of two and twenty, living with her parents in a dull, sober, well-conducted house in Knightsbridge, Kentish Town, or perchance in Newington Butts. She has no immediate prospect of being married, and has been getting rather thinner for the last year or two. She has occasionally some cough and expectoration, and is troubled with a stitch in her side;—nay more, she has a fair skin and blue eyes, and is therefore convicted, on the evidence of a thousand novels, of being in a consumption.

'Really Loo, my dear, that little hacking cough of yours quite alarms me; you positively *must* go to Mr. Long; I'm told he's very clever in these complaints.'

Now the truth is, that Miss Louisa's complaints are simply nervous or hysterical; and any thing capable of changing the hypochondriac current of her ideas, or relieving the dull monotony of her existence, will effect a temporary cure, be the remedy agreeable or disagreeable, be it prescribed by Maton or inflicted by Long;—thus marriage, a trip to Brighton, or a legacy of 10,000*l.* will prove as effective as assafœtida, a residence in a disturbed Irish district, reading Mr. Long's book, or being flayed by his liniment.

You see, my dear public, I have no spite against this famous liniment; quite the reverse; I believe it to be a good, scorching, burning, skin-destroying, slough-creating liniment, as one might wish to see on a summer's day—as good a liniment as the healing god might have used, when whilome in Harley-street—I beg pardon, in Phrygia—he cured Marsyas of all his ailments in one single visit. Nay, more, I must freely confess that there is one quality in which this vigorous liniment

> 'stands alone,

> Like Adam's recollection of his fall;'

a quality to which a red-hot poker, and sulphuric acid, and the cat-o'-nine-tails, and caustic potash, and other of its elder-born brothers, make no pretension—I mean its acute discriminating power. This is not one of those vulgar embrocations that fasten on friend and foe alike—one of those hot-headed inconsiderate lotions that would treat the skin behind which the lungs of Stentor were entrenched, like the skin of a Hawkes or a Fennel;—no, no; a liniment educated in the polite air of Harley Street would scorn the sordid manners of its relations brought up in hospitals, whose unfashionable site I am almost ashamed to mention. This sharp and super-human discernment will certainly place the liniment far above the stethoscope—no man will content himself with looking, or rather hearing, when he can look and cure at the same time. It was long ago said—

'Segnius irritant animos demissa per aures
Quam quæ sunt oculis subjecta fidelibus.-- *Hor.*

That is—

When St. John's learned eyes
Can read your chest and neck,
What man of sense would prize
The tube of Laennec ?

How curious it must be to observe a broad excoriation, the telegraphic signal of some vast abscess within, and to contrast it with the minor and interrupted flayings that indicate the presence of tubercles !"

The verse on Long and Laennec, in the foregoing extract, would puzzle all the lake poets of the present century, to reduce it to any standard of song, even in these days of irregular and eccentric versification ! We have not the most distant idea of the name of the author—but we strongly advise him to remain anonymous.

---

XXVI.

